# Treatment, Diagnostic Criteria and Variability of Terminology for Lateral Elbow Pain: Findings from an Overview of Systematic Reviews

**DOI:** 10.3390/healthcare10061095

**Published:** 2022-06-14

**Authors:** Luigi Di Filippo, Simone Vincenzi, Denis Pennella, Filippo Maselli

**Affiliations:** 1 Medicine Department, University of Rome “Tor Vergata”, 00133 Rome, Italy; fisioanalysis@gmail.com (L.D.F.); vincenzi.simone@gmail.com (S.V.); denispennella@gmail.com (D.P.); 2 FisioAnalysis Mædica, 15121 Alessandria, Italy; 3 Department Human Neurosciences, Sapienza University of Rome, 00185 Rome, Italy; 4 Centro Moove, 47042 Cesenatico, Italy; 5 Manual Therapy Lab Clinic, 70123 Bari, Italy; 6 Department of Medicine and Health Science “Vincenzo Tiberio”, University of Molise, 86100 Campobasso, Italy; 7 Sovrintendenza Sanitaria Regionale Puglia INAIL, 70126 Bari, Italy

**Keywords:** tennis elbow, lateral epicondylitis, lateral elbow tendinopathy, treatment, diagnosis

## Abstract

Background: Lateral elbow pain (LEP) represents a musculoskeletal disorder affecting the epicondyloid region of the elbow. The terminological framework of this problem in literature, to date, is confusing. This systematic review (SR) aims to analyse the panorama of the scientific literature concerning the pathogenetic framework, treatment, and clinical diagnosis of LEP. Methods: We conducted an SR according to the guidelines of the PRISMA statement. We performed research using the electronic Medline, Epistemonikos, and Cochrane Library databases. The research started on 12 January 2022 and finished on 30 April 2022. We included all systematic reviews and meta-analyses published, in English, between 1989 and 2022. The articles’ selection was based on critical appraisal using Amstar 2. In the selected reviews we obtained the etiopathogenic terminology used to describe the symptoms, treatment, and diagnostic criteria of LEP. Results: Twenty-five SRs met the eligibility criteria and were included in the study. From these SRs, 227 RCT articles were analysed and different treatments proposals were extracted, such as exercise, manipulation corticosteroid injection, and surgery. In the selected articles, 10 different terms emerged to describe LEP and 12 different clinical tests. The most common treatments detected in this SR were a conservative multimodal approach (e.g., eccentric exercises, manual therapy, acupuncture, ultrasound), then surgery or other invasive treatments (e.g., corticosteroid injection, tenotomy). The most common term detected in this SR was “lateral epicondylitis” (n = 95, 51.6%), followed by “tennis elbow” (n = 51, 28.1%) and “lateral epicondylalgia” (n = 18, 9.4%). Among the diagnostic tests were painful palpation (n = 101, 46.8%), the Cozen test (n = 91, 42.1%), the pain-free grip-strength test (n = 41, 19.0%), and the Maudsley test (n = 48, 22.2%). A total of 43.1% of RCTs (n = 96) included subjects with LEP > 3 months, 40.2% (n = 85) included patients with LEP < 3 months, and 16.7% of the items (n = 35) were not specified by the inclusion criteria on the onset of symptoms. Conclusions: In this SR, a considerable terminological heterogeneity emerged in the description of LEP, associated with the lack of clear and recognised diagnostic criteria in evaluating and treating patients with lateral elbow pain.

## 1. Introduction

Lateral elbow pain (LEP) represents a musculoskeletal condition, between musculoskeletal disorders (MSDs), affecting the epicondyloid region of the elbow [1,2]. It mainly affects workers between 35 and 54 years of age, with a prevalence of between 1% and 3%. The incidence in general practice is about 4–7 people per 1000 patients per year, producing a heavy economic burden due to loss of productivity, high costs for access to care services, and the impossibility of carrying out one’s work, which can last for several weeks [3,4].

Repetitive movements of flexion–extension and prone–supination of the elbow, lifting of heavy loads, and frequent extensions of the wrist against resistance contribute to the onset of this pathology, leading the person to reduced participation in daily life activities [5,6].

The management of lateral elbow pain has been a real challenge for clinicians and researchers for years, although several studies have been produced over the years on the treatment and mechanisms of this condition [7]. Several conservative treatments have been proposed, such as eccentric exercise and manual therapy. Corticosteroid infiltrations and platelet-rich plasma (PRP) showed efficacy in the short term but led to worse long-term results when compared to exercise [7].

Despite the high number of studies in the literature, LEP is a very recurrent pathology to date: One out of three people, after 12 months, still complained of painful and disabling symptoms despite rehabilitative treatment, and up to 5% of patients considered surgery [7,8].

These data suggest investigating the aetiology, diagnosis, and treatment of this musculoskeletal disorder to improve its clinical management.

In the literature, to date, the terminological framework of this problem is confusing. Indeed, researchers adopted more than 10 names (i.e., lateral elbow tendinopathy, lateral epicondylitis, tennis elbow, lateral epicondylalgia, lateral epicondylosis) to define LEP [9,10,11].

A tendon-centred etiological and diagnostic framework was perceived for the term “lateral elbow tendinopathy” (LET) [9,10].

The diagnosis of LET is based on an accurate medical history and the administration of specific tests to provoke the patient’s symptoms. For a correct differential diagnosis, clinicians should exploit imaging like ultrasound (US) and magnetic resonance imaging (MRI) [7,12].

However, in the literature, no reliable and validated test cluster allows for the identification and categorization of subjects with LEP to set up an effective therapeutic plan and reduce failures [8,13].

Only one primary study, with limited sample size, analysed the diagnostic accuracy of the Cozen and Maudsley tests, the two most commonly used provocative tests in diagnosing lateral elbow pain [8].

Coombes et al. [7], in 2015, were the first authors to propose a preliminary algorithm to guide clinicians in identifying different subgroups of patients with LEP and facilitate the decision-making process regarding the treatment choice. The subgrouping of patients with LEP was based on the analysis of prognostic factors (i.e., the severity of pain, disability, the presence of central sensitisation, neuromuscular impairments) and specific self-assessment questionnaires.

Numerous systematic reviews (SR) of LEP have been produced in recent years but none focus precisely on diagnostic labels and specific tests. The main goal is to “tidy up” this widespread and disabling condition to identify in future valuable tools to create a precise and reliable algorithm for the management of subjects with LEP, and moreover, to clarify whether the current terminologies used are accurate. An overview of SR represents a logical next step to provide decision-makers in healthcare and summarize the results generated by the SRs relating to a given topic [14].

This overview of SRs analyses the panorama of the scientific literature concerning the pathogenetic framework, the clinical diagnosis, and treatments of LEP. In particular, we considered the inclusion criteria and clinical tests used to identify patients with LEP within the studies and the terminology adopted to describe the symptomatology.

In light of what has been described in the literature to date, it would be appropriate for the authors to propose an “umbrella” term that includes different clinical pictures attributable to LEP and embraces the terminology, classification, and management.

In this umbrella term, specific subgroups of patients with lateral elbow pain should be considered, for which it would be helpful to propose the use of more current diagnostic tools for a clinical setting that helps to more carefully determine, where possible, the structure predominantly involved in the development of signs and symptoms, and define therapeutic paths according to different prognoses, capable of guiding clinicians towards more appropriate treatments for the patient, taking into account their relative impact on psycho-social aspects.

## 2. Methods

This overview of SRs was prospectively registered in PROSPERO (CRD42021266790) [15].

### 2.1. Study Design

The Preferred Reporting Items for Systematic Reviews and Meta-Analysis (PRISMA) protocol was used to design and report the present SR of SRs [16].

Moreover, due to the overviews of systematic reviews being relatively new approaches to synthesising evidence, the methods were described in the literature, and relevant research was used to plan the present overview [15].

### 2.2. Purpose of the Study

The following question was defined: “What are the most commonly used treatments, etiopathogenetic terminologies, and diagnostic criteria adopted to identify patients with LEP?”

### 2.3. Search Strategy

We performed research on the three main electronic databases for systematic reviews: Medline (1996–2021), the Epistemonikos Database (2009–2021), and Cochrane Library (1996–2021).

The keywords used for the purposes of the research were: “tennis elbow” (MeSH terms), “lateral epicondylitis,” “lateral elbow tendinopathy,” and “lateral epicondylalgia.” The Boolean operator OR allowed for the selection of most of the target SR. PubMed Clinical Queries was used on PubMed Central as a tool to enter the query string and select the systematic reviews and the meta-analysis on the topic.

This step was finally completed and integrated with manual research of the bibliographic references. The research started on 12 January 2022 and ended on 31 January 2022.

We have reported the complete search strategies in Appendix A.

### 2.4. Eligibility Criteria

We included articles according to the following criteria: (a) published from 1989 to 2022, (b) written in English, (c) relating to the diagnosis and treatment (conservative and surgical) of LEP, (d) defined one or more terms of classification of LEP, and (e) reported a study design as SR with or without metanalysis (MA).

We excluded articles published before 1989. RCTs, scoping reviews, literature reviews, and case studies were not included in our SR. Studies that were not exclusively about LEP were excluded.

### 2.5. Study Selection

Two reviewers (SV and DP) performed the selection and data collection process under the supervision of a third author (FM). First, all records were screened by the management software for Rayyan systematic reviews (https://rayyan.qcri.org, accessed on 14 January 2022), whereas references were managed by the Mendeley software (https://www.mendeley.com, accessed on 31 January 2022). Then, after removing the duplicates, the titles and abstracts were screened. Lastly, full texts of the identified studies were obtained for further assessment and analysed independently according to the eligibility criteria by two reviewers (S.V. and D.P.). Where appropriate, the authors were contacted in order to obtain the full text.

The language did not pose any barrier to the analysis of the articles, and a native US translator was consulted when necessary.

### 2.6. Data Collection

For each SR, the following data were extracted: study design (SR o SR with MA); author, year of publication; the number and characteristics of participants/populations; treatments; definition and/or any diagnostic criteria for LEP (e.g., specific diagnostic test or a diagnostic cluster); analysis of the variables and the outcome of the studies; and study settings/country.

Overlapping of RCTs within each SR was considered to avoid entering study data twice in the data analysis, as suggested from other overviews of SRs [15].

### 2.7. Quality Assessment

Furthermore, to make sure no methodological low-quality publications were included, only systematic reviews or metanalyses were selected through AMSTAR II (Assessing the Methodological Quality of Systematic Reviews) [17] and then analysed with the RoBis tool (Risk of Bias in Systematic Reviews) [18].

Although AMSTAR 2 is not aimed at generating an overall score cut-off, to judge the quality of the SRs, it recommends defining critical items, which helps identify any weaknesses in these items to make an overall assessment of the reliability of the results of the selected SRs. All SRs that received a low or very low overall rating (one point or more of a critical point) were excluded [17].

AMSTAR II is the most widely used SR assessment tool but focuses on the overall critical assessment of reviews and does not analyse the risk of bias in the systematic review. RoBis’s approach aligns with the most recent methods used to develop the risk of bias assessment tools and promises to improve the assessment process in the overview and guidelines. A recent study evaluated the interrater reliability (IRR) of AMSTAR II and RoBis in judging individual domains and the general methodological quality/risk of bias of systematic reviews, the concurrent validity of the instruments, and the time needed to apply them. The results showed that AMSTAR II and RoBis have overlapping IRRs, though they are different in construction and applicability [19].

According to the specific study design, the risk of bias (RoB) of the included studies was analysed using the RoBis tool [18]. The RoBis tool was used by two independent reviewers (S.V. and L.D.F.) to assess the risk of bias in selected SRs. All the studies included RCTs of a methodologically medium–high quality level that considered subjects displaying unilateral LEP. The score of the RoBis was not adopted as a criterion to include or exclude studies in this SR.

### 2.8. Agreement

Cohen’s Kappa (K) was used to assess the interrater agreement between the two authors (S.V., D.P.) for full-text selection (K = 0.78; 0.61–0.80 IC 95%). Cohens’ K was interpreted according to Altman’s definition: k < 0.20 poor; 0.20 < k < 0.40 fair; 0.41 < k < 0.60 moderate; 0.61 < k < 0.80 good; and 0.81 < k < 1.00 excellent [20].

### 2.9. Data Analysis

A standardized set of data was extracted for the selected SRs. Data collected included study design, sample characteristics, inclusion/exclusion criteria, participants, number of RCTs included, intervention, outcome, risk of bias, limits, and author’s conclusion. In addition, the terminology used to describe LEP was extracted from the studies.

A statistician implemented statistical data elaboration by using Excel^©^ (Microsoft, Washington, DC, USA, 2019) calculation sheets.

Data related to the internal and external validity of each selected study were also retrieved based on the RoBis criteria for quality assessment purposes.

## 3. Results

### 3.1. Study Selection Process

Electronic database searches yielded 118 SRs and 26 MAs about LEP.

After leaving out six duplicates, the articles underwent screening of pertinence according to the title and the abstract. Forty-eight articles were eliminated due to non-pertinence to the topic title.

**Figure 1:**
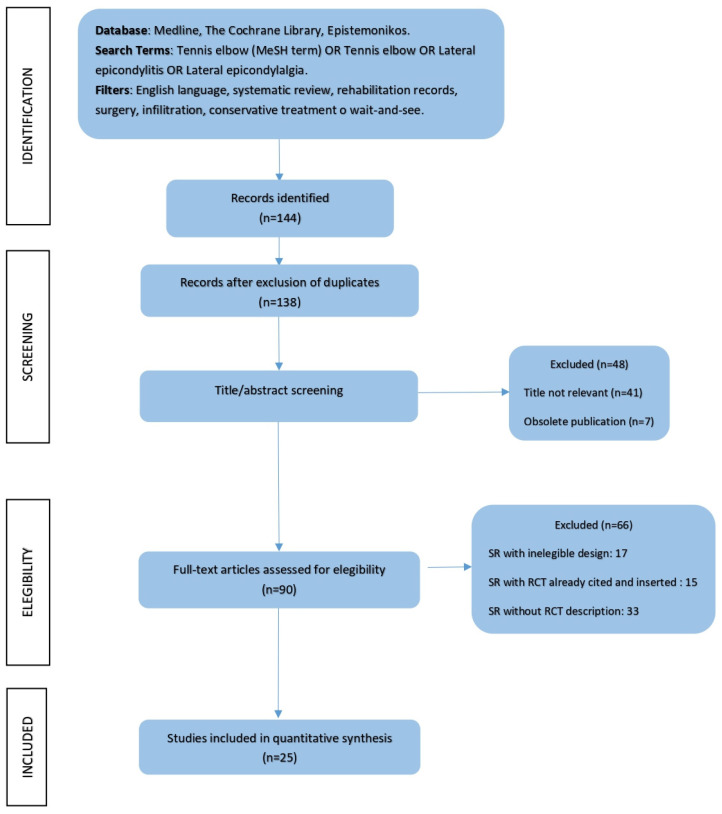
PRISMA flow diagram summarising the study selection process [16].

The 90 remaining full texts were analysed and underwent eligibility criteria of the AMSTAR 2 checklist [17]. Sixty-six articles were left out because the authors did not adequately describe the included studies (setting study and research design). The systematic reviews that did not provide a detailed description of the studies’ selection and the included criteria were not considered. The full-text analysis was carried out by two not-blinded editors (S.V. and D.P.); for further counsel, a third editor (L.D.F.) was asked for advice (Figure 1).

The research and the following selection of the studies led to the inclusion of 19 SRs and 6 SRs with MAs for 25 selected articles (Table 1).

### 3.2. Characteristics of Treatment and Patients

Within the included SRs, a range of the most used clinical treatment strategies in the case of LEP was analysed, among which were ultrasound therapy, the use of splints, acupuncture, extracorporeal shockwave therapy (ESWT), manipulations, low-level laser therapy (LLLT), therapeutic exercise, dry needling, surgery, PRP, corticosteroid injections, and the use of nonsteroidal anti-inflammatory drugs (NSAIDs). Two hundred sixteen RCTs of the respective SRs were examined. All the selected studies were approved by the ethical committee and the participants’ written informed consent was obtained. One of the 24 SRs analysed the efficacy of ultrasounds to treat LEP and comprised four RCTs [21]. Two reviews dealt with the use of splints in the case of elbow pain [22,24], whereas a 2004 Cochrane SR studied the effects of ESWT [26]. A 2004 review [31] analysed the efficacy of LLLT, and three SRs gathered the RCTs on therapeutic exercise [25,34,37,45]. Acupuncture in LEP treatment was studied in three SRs, including 16 RCTs of good methodological quality [23,38,40]. Two SRs collected RCTs that assessed the efficacy of manipulations in the case of LEP [27,42]. Within seven SRs, the authors pointed out a reduction of symptoms in subjects affected by LEP thanks to cortisone, botulinum, PRP injection, or the intake of NSAIDs [29,30,32,35,39,43]. Two SRs studied the most resolutive surgery techniques for LEP [33,41]. One study analysed the effect of trigger-point dry needling in subjects with LEP [44].

A total of over 20,000 patients were involved in the 227 RCTs analysed in this SR. For each study, the data linked to the terminology used to define the LEP and the inclusion criteria of the subjects were collected (symptom inception and clinical diagnostic tests). Details of the treatments and terminology adopted are described in Table 2.

### 3.3. Characteristics of the Included Studies

The oldest SR dated back to 1999 and analysed the effectiveness of US in LEP; the most recent SR was published in 2021 and collected the RCTs concerning the effectiveness of trigger-point dry needling in LEP [21,45]. The study by Bisset et al. collected the largest number of RCTs (24) and presented the largest number of selected subjects (1760) [27]. Butchbinder’s SR on the effectiveness of ESWT in the treatment of LEP selected only two high-quality RCTs [26].

Of 25 SRs, nine articles were related to a surgical or pharmacological approach (NSAIDs, corticosteroids, or PRP) in the case of LEP [28,29,30,32,33,35,36,39,43].

### 3.4. Risk of Bias of the Included Studies

Details of the RoBis of the included studies are presented in Table 3. Most items of the RoBis assessment tools used for the quality assessment were rated as low risk. Six SRs showed a low risk of bias in the items concerning the eligibility criteria, selection of studies, data collection, study appraisal, and synthesis [22,23,27,33,38,42]. Some SRs did not clearly describe the eligibility criteria based on the study characteristics and sources of information [26,30,37,39,43]. Items related to a low risk of bias in the selection of studies were lacking in seven SRs; in particular, problems were found concerning the research strategy used and the selection criteria [24,27,34,35,36,39,42]. Moreover, in four studies the item about the data collection and study appraisal was not completely described or had biases of assessment [26,29,37,41]. Synthesis and findings were evaluated. A high risk of bias in the synthesis and findings process was recorded in nine SRs, particularly in the presentation of the results and in the analysis of the selected studies [21,24,25,28,31,34,36,40,41,45].

### 3.5. Summary of Findings

**Figure 2:**
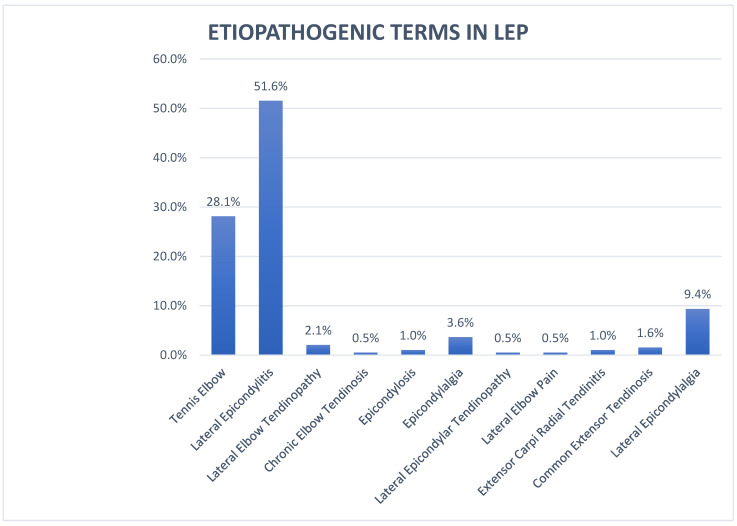
Terminology for lateral elbow pain (LEP); legend: LEP—lateral elbow pain.

**Figure 3:**
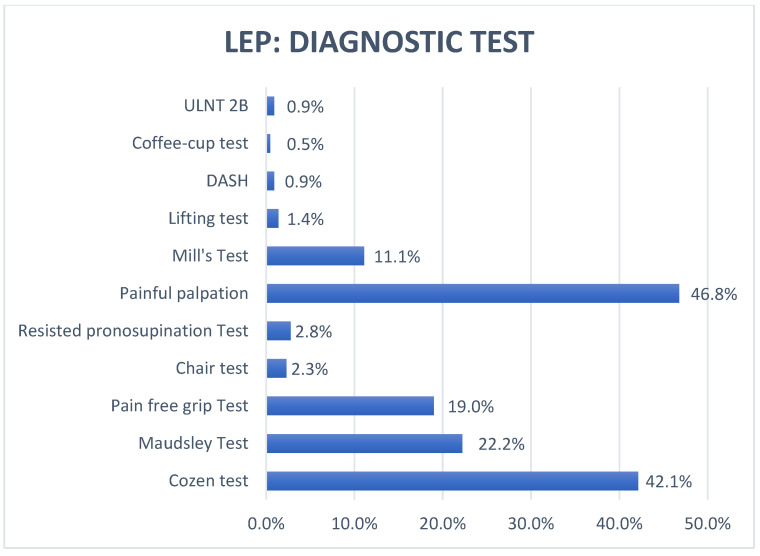
Diagnostic test in lateral elbow pain (LEP); legend: ULNT—upper limp neural test; DASH—Disabilities of the Arm, Shoulder, and Hand; LEP—lateral elbow pain.

**Figure 4:**
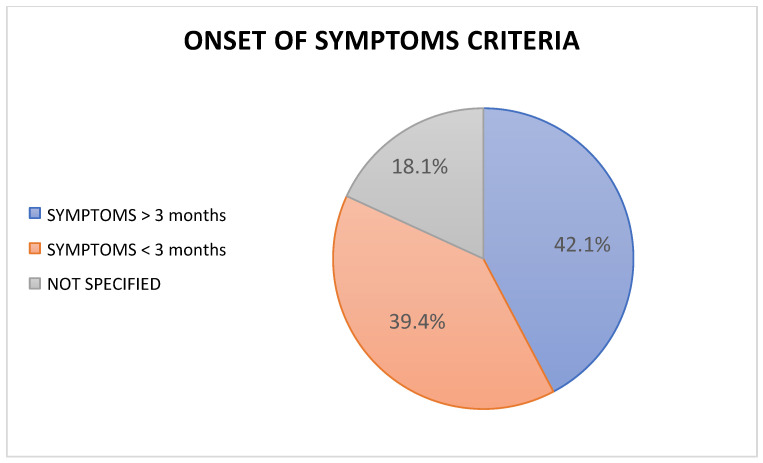
Onset of symptom criteria for LEP reported in the selected studies.

Results concerning the terminology, diagnostic test, and onset criteria of symptoms used in the selected articles are reported in Figure 2, Figure 3, and Figure 4.

### 3.6. Etiopathogenetic Terms in LEP

In the 25 SRs the symptomatology was described through the terms “tennis elbow”, “lateral epicondylitis”, “lateral elbow pain”, “lateral elbow tendinopathy”, “lateral epicondylalgia”, “epicondylosis” and “epicondilopathy” [21,22,23,24,25,26,27,28,29,30,31,32,33,34,35,36,37,38,39,40,41,42,43,44,45].

In the 227 RCTs collected through the selection of the 24 SRs, 11 different terms were recorded describing the same LEP clinical condition. The most used term was “lateral epicondylitis” (51.6%), followed by “tennis elbow” (28.1%) and “lateral epicondylalgia” (9.4%). Further terms used to define LEP from an etiopathological point of view were “epicondylalgia” (3.6%), “epicondylosis” (1.0%) “lateral elbow tendinopathy” (2.1%), “chronic elbow tendinosis” (0.5%), “common extensor tendinosis” (1.6%), “lateral epicondylar tendinopathy” (0.5%), “extensor carpi radial tendinitis” (1.0%), and “lateral elbow pain” (0.5%) [21,22,23,24,25,26,27,28,29,30,31,32,33,34,35,36,37,38,39,40,41,42,43,44,45].

Ninety-nine out of 227 articles used the term “lateral epicondylitis,” whereas 56 RCTs talked about “tennis elbow” and 18 about “lateral epicondylalgia.” Only one of the selected articles described the symptomatology as “lateral elbow pain” [33].

### 3.7. Including Criteria in LEP

Inclusion criteria of the subjects affected by LEP in the RCTs in the 25 SRs were examined. Painful palpation of the epicondyle turned out to be the most used clinical way to diagnose LEP (46.8%); however, more than half of the articles did not take it into account [24,25,27,29,30,31,34,35,36,43,44,45]. Considering the 227 RCTs, the Cozen test was one of the most recurring provocation tests in the literature (42.1%), whereas the Maudsley test appeared in 48 RCTs (22,2%). In 43 selected articles, the Cozen test was not combined with the Maudsley test to diagnose the presence of LEP [21,25,27,28,29,30,31,32,33,36,37,38,39,40,41,42,43]. The pain-free grip-strength test (PFGST) was used in 19% of the articles, meaning that this test was not considered throughout the diagnostic process in 168 out of 227 articles [21,22,24,25,26,27,28,29,31,33,34,35,36,37,38,41,45]. The Mill’s test was mentioned in 11.1% of the RCTs and was always combined with at least one other provocation test [21,26,28,30,31,32,34,36,37,38,39,42,43,44,45]. Further provocation tests were identified, among which were the chair test (2.3%), the resisted pronosupination test (2.8%), the lifting test (0.9%), and the coffee-cup test (0.3%). Two articles (1%) integrated the DASH scale (Disabilities of the Arm, Shoulder, and Hand) to diagnose LEP, although this tool is an upper-limb disability scale. The ULNT2B (upper limb neural tension test) was added in the diagnostic process only in two RCTs (0.6%) [21,44].

The onset timings of symptoms in the subjects selected by the 227 RCTs included in the SRs were collected. A total of 42.1% of the studies analysed patients that had been displaying symptoms for more than 3 months. A total of 89 articles (40.2%) included patients with LEP for less than 3 months. The remaining 39 articles did not describe a cut-off related to the timing of the symptoms’ onset [21,22,23,24,25,26,27,28,29,30,31,32,33,34,35,36,37,38,39,40,41,42,43,44].

## 4. Discussion

The data collection of the terms used to give a more aetiologically correct meaning to a set of clear symptoms such as the ones associated with LEP showed a wide heterogeneity dictated both by a merely biomedical view of the musculoskeletal problem and by the habit of using a term that was first coined in 1883 and is still used to this day [46]. The term “tennis elbow” was used in 56 out of the 227 selected RCTs and, indeed, refers to pathology with a high incidence in tennis. However, it is well known that LEP mainly affects the working population category, especially those involved with heavy lifting and subtle movements of the upper limbs; the LEP incidence in tennis is rather low and affects mainly amateur tennis players [5,12,14,46].

Evaluation using the RoBis tool showed a low bias risk in most of the selected SRs [18,19]. The studies showed good methodological rigour from the point of view of the eligibility and selection criteria of the RCTs. In contrast, the synthesis of the studies was often difficult due to the heterogeneity of the outcomes and the evaluation criteria.

### 4.1. Terminology Variability in LEP

The term “lateral epicondylitis” was used in 51.6% of the articles and recalled the primarily inflammatory nature of LEP. After several histopathological studies on tendons, Khan et al. (2002) recommended opting for the term “tendinopathy” to describe the symptomatology more accurately [47]. Indeed, several authors underlined that LEP is not caused by an inflammatory component but by a degenerative tendon process in the following years [46,47,48].

According to these studies, terms such as “epicondylosis” (1.0%), “chronic elbow tendinosis” (0.5%), “common extensor tendinosis” (1.6%), “lateral epicondylar tendinopathy” (0.5%), and “extensor carpi radial tendinitis” (1.0%) should be abandoned because they are obsolete.

The term “tendinopathy,” in particular “lateral elbow tendinopathy” or “lateral epicondylar tendinopathy,” was mentioned in five articles (2.1% and 0.5%). It is worth noting that three of these RCTs were selected from an SR by Raman et al. (2012) that dealt with the therapeutic exercise’s efficacy in the treatment of LEP [34]. This link between exercise and tendinopathy is tightly connected to the cultural revolution in the rehabilitation in treating overload issues and tendinopathies. Several authors, such as Maffulli, Cook and afterwards Rio, were among the first to understand the importance of exercise in this kind of pathology [49,50,51].

Among the 227 selected RCTs, the term “lateral epicondylalgia” was included in 19 studies (9.4%). In an SR by Bisset et al. (2005), 28 RCTs were analysed that dealt with the most effective interventions in the case of “lateral epicondylalgia” [27]. This terminological and conceptual transition underlines the shift from the biomedical pathology to the musculoskeletal disorder related to the bio–psycho–social sphere [11,27,52].

### 4.2. Classification Based on Onset of Symptoms

The psycho–social sphere seems to have a key role in developing the central sensitisation (CS) phenomenon in subjects with LEP. The CS phenomenon is directly associated with the onset of a disturbance in the musculoskeletal structure (low-back pain, cervicogenic headache, LEP) and is more recurring in subjects showing symptoms for three months or more [53]. In the inclusion criteria of the 227 RCTs, only 42.1% of the studies included patients with symptoms lasting more than three months. That means that the remaining articles (39.4% with symptoms < 3 months and 18.1% with non-specified temporal criteria) structured the RCTs based on a group of patients that was not homogeneous for the symptoms’ onset, the related psychosocial associations, and, therefore, the presence or not of the CS phenomenon. That could have modified the response of some subjects to the proposed interventions, causing a background bias that invalidates the quality and the results of the study itself.

### 4.3. Diagnostic Test in LEP

The literature-based and clinical approach mainly uses provocation tests that exacerbate the epicondylar pain to diagnose in the case of suspected LEP.

In this SR, eight different provocation tests were identified (painful palpation, Cozen test, Maudsley test, chair test, resisted pronosupination test, Mill’s test, lifting test, coffee-cup test): Painful palpation of the epicondyle (46.8%), the Cozen test (42.1%), and the Maudsley test (22.2%) were the most mentioned ones in the 216 selected RCTs.

The Cozen and Maudsley tests are the only ones that were submitted to a primary study that calculated their psychometric values. In particular, these two tests showed high sensitivity values (Cozen test, 84%; Maudsley test, 88%) [18]. Furthermore, clustering these two tests might further increase their reliability. Literature about psychometric values of other tests is quite weak, so they should be studied further. The PFGST was administered in 19.0% of the cases and represents a valid instrument for the assessment of the load’s tolerance and the excitability of the elbow with a high reliability (ICC > 0.97) and a minimal clinical importance difference (MCID) of 7 kg, representing an 18% change of the mean normative value [27,45,54,55]. ULNT2B was proposed in two RCTs included to analyse the presence of an entrapment of the radial nerve at the level of Frohse’s arcade or inside of the brachioradial muscle [56,57]. In this regard, other tests have been proposed in the literature to evaluate the presence of peripheral nerve entrapment in LEP, such as the Rule-of-Nine test [58].

Most of the tests mentioned in the studies aim to elicit a symptom that recalls an insertional tendinopathy of the extensors of the wrist and fingers, but they do not allow for a differential diagnosis with other causes of LEP such as radial nerve entrapment and intra-articular pathology.

In fact, the studies showed that several cases of persistent LEP were related to a high incidence of intra-articular alterations caused by a condition of ligamentous micro-instability at the radial capitellum level. (S.M.I.L.E., symptomatic minor instability of the lateral elbow) [59,60]. Several cases of LEP revealed a ligamentous laxity of the radial component of the radial collateral ligament and/or of the radial annular ligament that lead to phenomena of synovitis and/or chondropathy at the radial capitellum level (CLAC lesion, “chondropathy of the lateral aspect of the capitellum”). From a diagnostic and prognostic point of view, it would be useful to start considering the intra-articular issue in the assessment of LEP to create a reliable and efficient procedural algorithm for identifying the main pain generator and for the treatment of this symptomatology. This kind of approach would allow for different management of cases of persistent LEP that have not shown improvement after the suggested rehabilitative treatment and that still show disability and restrictions in movement.

### 4.4. Lateral Elbow Disorders: A New Proposal

Following the previous paragraphs, we believe that the time has come to find, through a common language, a definition to better describe the disorders of the lateral part of the elbow. According to the authors, lateral elbow disorders (LEDs) seems the most appropriate term to describe them. Furthermore, we want to propose the use of more current tools for a clinical setting, capable of identifying specific subgroups of LEDs that (A) help to determine more carefully the description of signs and symptoms of patients suffering from lateral elbow pain, (B) respond to different prognosis and outcome times of the proposed treatments and their relative impact on the psycho–social aspects of the patient, and (C) in the presence of a high predominance of yellow flags, the patient should be monitored and educated, thus modifying any dysfunctional beliefs and overestimated expectations about elbow pain and reconceptualising, on a cognitive level, any fear, harm, and avoidance about elbow activity [61]. In this regard, we believe it is fundamental that the clinical framework follow a rational construct capable of:

(1) Analysing any red flags to analyse the presence of situations that imply a non-musculoskeletal problem. In this case, the patient needs to be referred to the doctor for the most appropriate diagnostic investigations (screening for referral) [62,63,64,65];
(2) Guiding the clinician in determining whether there is a structure predominantly involved in and responsible for the lateral musculoskeletal disorder of the elbow (muscle–tendon, joint, neural) capable of influencing the prognosis and the type of treatment (conservative and/or surgical); and
(3) Recognising profiles of patients who, depending on the time of suffering, may present the risk of developing yellow flags capable of slowing down or altering the treatment process and/or deteriorating adherence to the therapeutic plan.

Following an evaluation algorithm proposed in a previous publication with the I-APPLEp algorithm [11], we believe that replacing the term “LEP” with “LEDs” is necessary. Our main goal is to describe and identify the lateral problems of the persistent-recalcitrant elbow—longer than three months—as an alternative to the old labels, among which are “epycondilitys,” “tennis elbow,” and “epycondilalgia.” Accordingly, we propose a modified version of the I-APPLE algorithm: LED-APP, the Lateral Elbow Disorders Approach (reported in Figure S1). It considers four possible subgroups of clinical pictures caused by three main anatomical complexes responsible for signs and symptoms, responding to different treatment principles and prognostic times. Namely, the algorithm includes the following as subgroups:

- T-LED (Lateral Elbow Disorders—Tendinopathic Prevalence);
- A-LED (Lateral Elbow Disorders—Arthropathic Prevalence);
- N-LED (Lateral Elbow Disorders—Neuropathic Prevalence); and
- M-LED (Lateral Elbow Disorders—Mixed Form).

The rationale for the new LED-APP assessment and treatment framework provides different steps of clinical assessment. In **Clinical Assessment 1,** we evaluate the involvement of the tendon component and the relative pain reported by the patient through the tendon load tests suggested by the literature as the most sensitive (rule out: Cozen test, Maudsley test, pain-free grip-strength test) [8,27]. The minimal clinical importance difference (MCID) of PFGS has been reported to be 7 kg in patients with LEP and represents an 18% change in the mean normative value of grip strength (38.4 kg) for men and women aged 40–50 years [27,45,54,55].

In case of positivity of these tests, we consider the clinical picture with tendinopathic prevalence (T-LED), and the treatment will be oriented towards desensitising pain with manual therapy and exercise and improving the load tolerance (load capacity) of the whole muscle–tendon system with progressive therapeutic exercise with a prognostic perspective of 4–6 weeks [66,67,68,69].

In case of failure of the conservative approach (NPRS <2 points, DASH <10 points, PRTEE <10 points), we consider it useful to refer patients to an orthopaedist. The main aim is to offer diagnostic imaging (Msk-US imaging, X-ray, MRI) and choose the most appropriate therapeutic path up to possible surgery [11]. In case of a negative Clinical Assessment 1, we will move on to **Clinical Assessment 2**, in which we evaluate whether the symptoms reported by the patient are attributable to joint pain through the direct provocation of the humero-radial joint structures with two provocative tests: SALT (supination antero-lateral pain test), proven to be sensitive and accurate for pictures of synovitis and anterior patolassity, and PEPPER (posterior elbow pain by palpation–extension of the radiocapitellar joint), proven to be sensitive and accurate for pictures of radial head chondropathy [70]. If these tests are positive (ad one of the two tests), the clinical picture will be considered a prevalent lateral elbow disorders (A-LED) with characteristics attributable to the clinical picture of SMILE (symptomatic minor instability of the lateral elbow) [59] and the treatment will be oriented to the protection of the joint component with the use of splints, braces and/or bandages, and manual therapy techniques of arthrokinematics [71] for pain control and intense therapeutic exercise with a prognostic outlook of 12–16 weeks [22,42,71]. In case of failure of the conservative approach (NPRS <2 points, DASH <10 points, PRTEE < 10 points), we consider it useful to refer patients to the orthopaedist. The main aim is to offer diagnostic imaging (Msk-US imaging, X-ray, MRI) and choose the most appropriate therapeutic path up to possible surgery [11].

In case of a negative Clinical Assessment 2, we will move on to **Clinical Assessment 3**, which assesses whether the patient’s symptoms are attributable to neural entrapment phenomena in Frohse’s arch (NIP syndrome or radial tunnel syndrome) or entrapment to the intermuscular septum between the extensor radial long carpus and the brachioradialis (Wartemberg syndrome) through clinical tests such as ULNT2b. If positive, proceed with two further tests: the Rule-of-Nines test and the Tinel test; in case of positivity to at least one of the two, the clinical picture will be considered a neural prevalence of lateral elbow disorder (N-LED) with the related sub-declinations. Thus, the treatment will include a manual therapy approach composed of neurodynamic and myofascial techniques associated with dynamic thermoplastic splints, ESWT, neurodynamic sliding or tensioning techniques, and dynamic stretching, with a prognostic perspective of (4) 8–12 weeks [72]. In case of failure of the conservative approach (NPRS <2 points, DASH <10 points, PRTEE <10 points), we consider it useful to refer patients to the orthopaedist. The main aim is to offer diagnostic imaging (Msk-US imaging, X-ray, MRI) and choose the most appropriate therapeutic path up to possible surgery [11].

In case of a negative Clinical Assessment 3, the signs and symptoms will be attributed to clinical pictures on a mixed basis (Mixed Form: M-LED), which should be approached and considered according to the clinical picture of reference. On the other hand, if the anamnestic collection and the description of the signs and/or symptoms are attributable to an uncommon specific problem, such as a possible expression of important and/or serious problems (e.g., trauma, fever, weight loss, infection, recent surgery, systemic disease, fracture, tumour), it is essential to refer back to the specialist for clinical and diagnostic study (screening for referral).

### 4.5. Limits

The SRs were selected through the RoBis tool to assess the risk of bias; this tool should not be used to generate a summary “quality score” because of the well-known problems associated with such scores. Therefore, the pooling of SR data does not present a cut-off for the quality screening of the articles. Inter review blindness was not maintained in the full-text analysis of the selected studies.

The authors are aware that the type of studies selected is not the best for evaluating a clinical condition’s taxonomy, classification, or diagnostic criteria. However, the choice to select systematic reviews of RCTs seemed a much more clinical and pragmatic choice to the authors. Since RCTs are the best studies to evaluate the best treatment, we started from this type of study to understand how these studies made diagnoses, which tests they relied on, and what they called these clinical conditions of LEP.

In future studies, this analysis of the literature could be helpful to analyse the reliability of clinical tests for patients with LEP and create a new algorithm of assessment and diagnosis. Lastly, quality studies to test its clinical efficacy are required to apply a structured and rigorous validation course.

### 4.6. Consistency

In the literature, to date, the terminological framework of this problem is confusing [73]. Therefore, there is a need for standard and internationally accepted definitions for LEP. LEP is defined as an overuse injury due to an unbalance between the resistance capacity of connective tissue and the biomechanical solicitations on the lateral aspect of the elbow [1,2,3,4,5,6,7,9]. Furthermore, pain is not the only symptom complained of by the patient, but is often associated with disabilities, functional impairments, and loss of social, sports, and work participation [9,10,11].

Therefore, in our view, a more suitable word may be “disorder” (lateral elbow disorders—LEDs), which better describes multifactorial conditions, which include, besides structural aspects, psychosocial elements often present in nonspecific painful disorders like LEP [5,6,9,10].

Our SR confirmed the findings for LEP, like in recent literature, which concluded that the evidence about the terminology of LEP is scarce and derived from studies not of good methodological quality [47,48,49,74,75]. This SR showed a quite high prevalence among studies of the term “LEP,” but this finding, albeit relative to a wider sample of patients, does not specifically include accurate reasons to adopt this term exclusively.

## 5. Conclusions

LEP includes a wide range of inflammatory and degenerative conditions affecting the muscles, tendons, ligaments, joints, peripheral nerves, and supporting blood vessels. These include clinical syndromes such as intra-articular and ligament disorders, tendinopathy, and peripheral nerve compression pathology.

In this SR, the terminological analysis of LEP that was carried out shows the need for “tidying up” within the wide range of terms and descriptions related to this symptomatology. The lack of clarity from a terminological point of view has led clinicians and researchers to define these symptoms with different terms, shifting from a traditional biomedical view to a biopsychosocial one, without determining a univocal term shared by everyone that could describe the pathology clearly and correctly. Furthermore, not even at the diagnostic level is there currently a validated and reliable cluster of tests in the literature that allows for the distinction between extra-articular-based symptomatology and an intra-articular-based one in a patient with LEP to define a suitable pathway of treatment.

## Figures and Tables

**Table 1:** Data extraction.

Review Year Country	Review Aim	Search Strategy	Studies and Participants	Patients, Interventions, Comparison, Outcome, and Study Type (PICOS)	Risk of Bias	Limits	Author’s Conclusion
**van der Windt et al.** **(1999)** [21] **The Netherlands**	To evaluate the effectiveness of ultrasound therapy in the treatment of musculoskeletal disorders and lateral epicondylitis (LE).	MEDLINE, EMBASE No search start date. Last search date in July 1997. Search terms defined. No limitation described. RCTs were screened. No evidence of reference checking. Eligibility criteria: patients with pain and/or restriction of range of motion associated with musculoskeletal disorders, RCTs, English.	RCT s= 4 N = 123	Population: patients with lateral epicondylitis Intervention: US Comparison: US sham, low-level laser, exercise Study type: RCTs	No evidence of quality assessment	Only RCTs in the US and LE were included.	The findings reported for lateral epicondylitis were less consistent and may warrant further evaluation.
**Struijs et al.** **(2002)** [22] **Australia**	To determine the efficacy of treatment of lateral epicondylitis by an orthotic device.	MEDLINE, EMBASE, CINAHL. No search start date. Last search date in 1999. Search terms defined. No limitations described. RCTs were screened. No evidence of reference checking. Eligibility criteria: patients with lateral epicondylitis of the humerus (tennis elbow), RCTs, English.	RCTs = 5 N = 300	Population: patients with lateral epicondylitis Intervention: orthotic devices Comparison: corticosteroid injection, anti-inflammatory cream, splintage, physiotherapy Study type: RCTs	Clear quality appraisal of the studies	A standard set of valid and reliable outcome measures should be incorporated into the RCTs.	No definitive conclusions can be drawn concerning the effectiveness of orthotic devices for lateral epicondylitis. More well-designed and well-conducted RCTs of sufficient power are warranted.
**Green et al.** **(2002)** [23] **Australia**	To determine the effectiveness of acupuncture in the treatment of patients with lateral elbow pain with respect to symptom reduction, including pain, improvement in function, grip strength, and adverse effects.	MEDLINE, EMBASE, CINAHL No search start date. Last search date in June 2001. Search terms defined. No limitations described. RCTs were screened. Evidence of reference checking. Eligibility criteria: lateral elbow pain and acupuncture, RCTs, English.	RCTs = 4 N = 239	Population: patients with lateral epicondylitis Intervention: acupuncture Comparison: sham acupuncture, low-level laser, vitamin B12 Study type: RCTs	Quality assessment completed but criteria and explanation unclear.	Trials should be adequately powered, attempt to blind both participants, including outcome measures of pain and function and adverse effects.	There is insufficient evidence to either support or refute the use of acupuncture (either needle or laser) in the treatment of lateral elbow pain.
**Borkholder et al.** **(2004)** [24] **USA**	To confirm or refute the efficacy of using splints in the treatment of lateral epicondylitis.	CINAHL, EMBASE, PEDro, and Cochrane databases. No search start date. Last search date in December 2003. Search terms defined. No limitations described. RCTs were screened. Evidence of hand searching. Eligibility criteria: splint and lateral elbow pain, RCTs, English.	RCTs = 8 N = 347	Population: patients with lateral epicondylitis Intervention: splinting Comparison: other splint, manipulation, anti-inflammatory cream, diclofenac Study type: RCTs	Clear quality appraisal of the studies.	Duration of symptoms was not considered in the majority of the included studies.	Early positive, but not conclusive, support for the effectiveness of splinting lateral epicondylitis.
**Trudel et al.** **(2004)** [25] **Canada**	To determine the effectiveness of conservative treatments for lateral epicondylitis and to provide recommendations based on this evidence.	CINAHL, EMBASE, PEDro, and Cochrane databases. Search start date 1983. Last search date in March 2003. Search terms defined. No limitations described. RCTs were screened. Evidence of hand searching. Eligibility criteria: English, adults (age 18+), humans, RCTs or quasi-RCTs, lateral epicondylitis, and rehabilitation.	RCTs = 21 N = 1666	Population: patients with lateral epicondylitis Intervention: US, acupuncture, rebox, wait-and-see, exercise, mobilisation, ionisation, laser, pulsed electromagnetic field Comparison: phonophoresis, sham, injection Study type: RCTs	Clear quality appraisal of the studies.	No adequate blinding measures, follow-up, and standardised outcome measures in RCTs.	There is a number of good-quality studies on various therapeutic interventions for lateral epicondylitis that demonstrate a variety of effective treatment options.
**Buchbinder et al.** **(2004)** [26] **Australia**	To determine the effectiveness and safety of shockwaves (ESWT) in the treatment of adults with lateral elbow pain.	MEDLINE, CINAHL, EMBASE, SCISEARCH, Cochrane Clinical Trials trial database. No search start and finish date. Search terms defined. No limitations described. RCTs were screened. No eligibility criteria.	RCT = 2 N = 372	Population: patients with lateral epicondylitis Intervention: ESWT Comparison: placebo ESWT Study type: RCTs	Quality assessment completed but criteria and explanation unclear.	Unclear allocation procedures in the trial of Rompe et al. and the treatment allocation of those who dropped out of the trial (13% of participants) was not reported.	The effectiveness of ESWT is unclear. The two trials included in this review yielded conflicting results. Further trials are needed to clarify the value of ESWT for lateral elbow pain.
**Bisset et al.** **(2005)** [27] **Australia**	To look at the effectiveness of physical interventions on clinically relevant outcomes for LE.	MEDLINE, CINAHL, EMBASE, Web of Science, Allied and Complimentary Medicine, SPORTDiscus, PEDro. Last search date in September 2003. Search terms defined. No limitations described. RCTs were screened. Eligibility criteria: RCT, English, patient with lateral elbow pain.	RCTs = 24 N = 1760	Population: patients with lateral epicondylitis Intervention: laser, ESWT, manipulation, mobilisation, exercise tape, orthotics, acupuncture, laser, iontophoresis Comparison: sham ESWT, sham tape, sham acupuncture Study type: RCTs	Clear quality appraisal of the studies.	Duration of symptoms and follow-up was not considered in the majority of included studies.	Evidence is accruing that does not support the use of ESWT, but there is indication for further research with long-term follow-up into manipulation and exercise as forms of treatment for LE.
**Herd,** **(2008)** [28] **The Netherlands**	To review the effectiveness of manipulation in treating lateral epicondylalgia.	MEDLINE, Cumulative Index of Nursing and Allied Health Literature, Health Source, SPORTDiscus, Physiotherapy Evidence Database. Search start date 1929. Last search date in November 2007. Search terms defined. No limitations described. RCTs were screened. No evidence of reference checking. Eligibility criteria: English, experimental design, subjects with lateral epicondylitis, manipulative treatment.	RCTs = 13 N = 639	Population: patients with lateral epicondylitis Intervention: manipulative therapy, Cyriax, MWM Comparison: exercise, injection, wait-and-see, US, friction massage Study type: RCTs	Clear quality appraisal of the studies.	Variability regarding manipulative technique, comparison interventions, follow-up, and outcome measures. Only one reviewer determined appropriateness for inclusion.	Current evidence supports Mulligan’s mobilisation with movement not only in providing immediate benefits but also improving outcomes at short- and long-term follow-up. A subgroup of patients with LE exists who would benefit from treatment directed at the cervical spine.
**Barr et al.** **(2009)** [29] **UK**	To compare the effectiveness of corticosteroid injections with physiotherapeutic interventions for the treatment of lateral epicondylitis (tennis elbow).	AMED, MEDLINE, CINAHL, EMBASE, Cochrane Central Register of Controlled Clinical Trials, Metaregister of Controlled Clinical Trials, PEDro. Search start date 1966. Last search date in March 2009. Search terms defined. No limitations described. RCTs were screened. Evidence of hand searching. Eligibility criteria: English, RCTs, lateral epicondylitis and corticosteroid injection.	RCTs = 3 N = 596	Population: patients with lateral epicondylitis Intervention: corticosteroid injection, corticosteroid injection with exercise and manipulation Comparison: exercise, US, wait-and-see, paracetamol, no treatment Study type: RCTs	Clear quality appraisal of the studies.	No follow-up in RCTs, no similar outcome measures.	Corticosteroid injections are effective at short-term follow-up, and physiotherapeutic interventions are effective at intermediate- and long-term follow-up. However, any conclusions drawn must be interpreted with caution.
**Coombes et al.** **(2010)** [30] **Australia**	To review the clinical efficacy and risk of adverse events of injections (including corticosteroids) for treatment of tendinopathy in the short term, intermediate term, and long term, and in different areas of tendinopathy.	MEDLINE, CINAHL, EMBASE, Web of Knowledge, Allied and Complementary Medicine, SPORTDiscus, Cochrane Controlled Trial Register, and Physiotherapy Evidence Database. No starting data search. Last search date in March 2010. Search terms defined. No limitations described. RCTs were screened. Evidence of hand searching. Eligibility criteria: RCTs, tendinopathy, and injection.	RCTs = 12 N = 1034	Population: patients with tendinopathy Intervention: corticosteroid injection, corticosteroid injection with exercise Comparison: physiotherapy, wait-and-see, NSAIDs, manipulation. Study type: RCTs	Clear quality appraisal of the studies.	No concealed allocation and similar outcome measures in the majority of included RCTs.	Despite the effectiveness of corticosteroid injections in the short term, non-corticosteroid injections might be of benefit for long-term treatment of lateral epicondylalgia. However, response to injection should not be generalised because of variation in effect between sites of tendinopathy.
**Tumilty et al.** **(2010)** [31] **New Zealand**	To assess the clinical effectiveness of low-level laser therapy (LLLT) in the treatment of tendinopathy.	MEDLINE, PubMed, CINAHL, AMED, EMBASE, All EBM (Evidence-Based Medicine) reviews, PEDro (Physiotherapy Evidence Database), SCOPUS. No starting data search. Last search date in August 2008. Search terms defined. No limitations described. RCTs were screened. No evidence of reference checking. Eligibility criteria: RCT, tendinopathy, and LLLT.	RCTs = 12 (10) N = 422	Population: patients with tendinopathy Intervention: LLLT Comparison: placebo, Tecar, friction massage, corticosteroid injection Study type: RCTs	Clear quality appraisal of the studies.	Only RCTs on LLLT and LE were included. Poor blinding procedures and reliable outcome measures.	LLLT can potentially be effective in treating tendinopathy when recommended dosages are used. The 12 positive studies provide strong evidence that positive outcomes are associated with the use of current dosage recommendations for the treatment of tendinopathy.
**Kalichman et al.** **(2011)** [32] **Israel**	To determine the efficacy of botulinum toxin for the treatment of chronic lateral epicondylitis.	PubMed, MEDLINE, CINAHL, Google Scholar, EMBASE, PEDro, ISI web of Science databases. No starting data search. Last search date in November 2009. Search terms defined. No limitations described. RCTs were screened. Evidence of hand searching. Eligibility criteria: botulinum toxin A for treatment of lateral epicondylitis, RCTs.	RCTs = 4 N = 278	Population: patients with lateral epicondylitis Intervention: botulinum toxin A injection Comparison: placebo (saline solution) Study type: RCTs	Clear quality appraisal of the studies.	No concealed allocation, description of adverse effect, and similar outcome measures in the majority of included RCTs.	Current literature provides support for use of botulinum toxin A injections into the forearm extensor muscles (60 units Disport or equivalent) for the treatment of chronic treatment-resistant lateral epicondylitis.
**Buchbinder at al.** **(2011)** [33] **Australia**	To determine the benefits and safety of surgery for lateral elbow pain.	CENTRAL (The Cochrane Library), MEDLINE, EMBASE, CINAHL, and Web of Science. Search start date 1966. Last search date in December 2010. Search terms defined. No limitations described. RCTs were screened. No evidence of hand searching. Eligibility criteria: lateral elbow pain and surgery.	RCTs = 5 N = 193	Population: patients with lateral epicondylitis Intervention: “open” surgery, percutaneous surgery Comparison: percutaneous surgery, botulinum toxin, ESWT Study type: RCTs	Clear quality appraisal of the studies.	Trials were susceptible to bias and hampered by inadequate reporting and small sample size.	Due to a small number of studies, large heterogeneity in interventions across trials, small sample sizes, and poor reporting of outcomes, there is insufficient evidence to support or refute the effectiveness of surgery for lateral elbow pain.
**Raman et al.** **(2012)** [34] **Canada**	To synthesise the quality and content of clinical research addressing type and dosage of resistance exercises in lateral epicondylosis	MEDLINE, EMBASE, CINAHL, SCOPUS. Search start date 1966. Last search date in December 2010. Search terms defined. Studies that investigated surgery, orthoses (splints), shock wave therapy, electrical stimulation, steroid injections, or casts were excluded. RCTs were screened. No evidence of hand searching. Eligibility criteria: lateral epicondylosis and exercise (strength, resistance, eccentric, concentric)	RCTs = 9 N = 697	Population: patients with lateral epicondylitis Intervention: resistance training (isometric, eccentric, concentric, isokinetic) Comparison: static stretching, ice, manipulation, forearm band; US Study type: RCTs	Clear quality appraisal of the studies.	Lack of high-quality trials that compared different exercise types or dosage, and a lack of detailed descriptions of exercise parameters in many published studies.	Strengthening using resistance exercises is effective in reducing pain and improving function for lateral epicondylosis, but optimal dosing is not defined.
**Ahmad et al.** **(2013)** [35] **UK**	To evaluate the evidence for the application of platelet-rich plasma (PRP) in lateral epicondylitis.	MEDLINE, EMBASE, CINAHL PubMed. Search start date 1966. Last search date in 2011. Search terms defined. No limitations described. RCTs were screened. No evidence of hand searching. Eligibility criteria: Human, RCTs, English, PRP in patients with lateral epicondylitis.	RCTs = 8 N = 507	Population: patients with lateral epicondylitis Intervention: PRP Comparison: saline injection, placebo Study type: RCTs	Clear quality appraisal of the studies.	Heterogeneity of patient population, variation of PRP preparation, and lack of standard outcome measures.	Limited but evolving evidence for the use of PRP in lateral epicondylitis; however, further research is required to understand the concentration and preparation that facilitate the best clinical outcome.
**Pattanittum et al.** **(2013)** [36] **Australia**	To assess the benefits and harm of topical and oral NSAIDs for treating people with lateral elbow pain.	MEDLINE, EMBASE, CINAHL, Cochrane. Search start date 1966. Last search date in 2011. Search terms defined. No limitations described. RCTs were screened. No evidence of hand searching. Eligibility criteria: Human, RCTs, NSAIDs in lateral elbow pain.	RCTs = 14 N = 938	Population: patients with lateral epicondylitis Intervention: NSAIDs Comparison: placebo Study type: RCTs	Clear quality appraisal of the studies.	RCTs did not provide enough published data, or did not provide data in a form that could be extracted for meta-analysis.	Limited evidence from which to draw firm conclusions about the benefits or harm of topical or oral NSAIDs in treating lateral elbow pain.
**Cullinane et al.** **(2014)** [37] **New Zealand**	To establish the effectiveness of eccentric exercise as a treatment intervention for lateral epicondylitis.	ProQuest, Medline via EBSCO, AMED, Scopus, Web of Science, CINAHL No starting search date. Last search date in 2011. Search terms defined. Limits: corticosteroid injections prior to the intervention or as part of the treatment or comparative therapy. RCTs were screened. No evidence of hand searching. Eligibility criteria: English, RCTs, tennis elbow, and eccentric exercise.	RCTs = 12 (3) N = 611	Population: patients with lateral epicondylitis Intervention: Eccentric exercise, eccentric exercise with other therapies Comparison: iontophoresis, US, stretching Study type: RCTs	Quality assessment completed but criteria and explanation unclear.	Lack of blinding of participants and treatment providers, lack of control group and standardised diagnostic criteria.	The majority of consistent findings support the inclusion of eccentric exercise as part of a multimodal therapy programme for improved outcomes in patients with lateral epicondylitis.
**Tang et al.** **(2015)** [38] **China**	To assess the effectiveness and safety of acupuncture for lateral epicondylitis (LE).	EMBASE, PubMed, the Cochrane Library, China National Knowledge Infrastructure (CNKI), Chinese Scientific Journal Database (VIP database), Wanfang Database, and Chinese Biomedical Literature Database (Sinomed). No starting search date. Last search date in 2015. Search terms defined. Limitations: no laser stimulation, no acupressure, no other type of acupuncture. RCTs were screened. No evidence of reference and hand searching. Eligibility criteria: RCTs, acupuncture, and lateral epicondylitis.	RCTs = 4 N = 309	Population: patients with lateral epicondylitis Intervention: acupuncture, electro-acupuncture Comparison: sham acupuncture, blockage therapy Study type: RCTs	Quality assessment completed but criteria and explanation unclear.	No detailed definition on random sequence generation, allocation concealment, and blinding of participants and personnel.	For the small number of included studies with poor methodological quality, no firm conclusion can be drawn regarding the effect of acupuncture on elbow functional status and myodynamia for LE.
**Tsikopoulos et al.** **(2016)** [39] **Greece**	To compare the efficacy of autologous whole blood with that of corticosteroid injections on epicondylopathy and plantar fasciopathy (PF).	PubMed, Web of Science, CENTRAL, and Scopus. No starting search date. Last search date on 6 May 2015. Search terms defined. No limitations described. Evidence of reference and hand searching. Eligibility criteria: Human, English, RCTs, autologous venous blood with that of corticosteroids on either epicondylopathy or PF.	RCTs = 9 (5) N = 447 (209)	Population: patients with lateral epicondylitis Intervention: autologous whole-blood intervention, corticosteroid injection Comparison: autologous whole-blood intervention, corticosteroid injection, placebo Study type: RCTs	Clear quality appraisal of the studies.	Eight RCTs were conducted in Asia. The follow-up in eight studies did not exceed six months.	Corticosteroids were marginally superior to autologous whole blood in relieving pain on plantar fasciopathy at 2–6 weeks. Autologous whole blood provided significant clinical relief on epicondylopathy at 8–24 weeks. Conclusions were limited by the risk of bias.
**Mattie et al.** **(2017)** [40] **USA**	To analyse currently available controlled studies on percutaneous tenotomy and its efficacy for the treatment of lateral epicondylitis.	MEDLINE, EMBASE, CINAHL, Cochrane, Web of Science. No starting search date. Last search date in November 2015. Search terms defined. No limitations described. Evidence of reference and hand searching. Eligibility criteria: English, RCTs, tennis elbow, and percutaneous tenotomy.	RCTs = 6 N = 242	Population: patients with lateral epicondylitis Intervention: percutaneous tenotomy Comparison: / Study type: prospective studies	Quality assessment completed but criteria and explanation unclear.	The included RCTs had a small sample size and patient self-selection for The procedure. The studies included variability of time in follow-up and in duration of symptoms.	Percutaneous tenotomy presents an alternative to surgical release of the common extensor tendon for the treatment of chronic tendinosis at the lateral epicondyle of the elbow. Current research supporting the efficacy of this procedure, however, is of low quality (level II to level IV).
**Burn et al.** **(2017)** [41] **USA**	To determine whether the choice of surgical technique (open, percutaneous, or arthroscopic) would lead to significantly different clinical outcomes in lateral epicondylitis (LE).	PubMed, Cochrane Central Register of Controlled Trials, and Google Scholar. No starting search date. Last search date in July 2016. Search terms defined. No limitations described. Evidence of reference and hand searching. Eligibility criteria: English, RCTs, tennis elbow, and surgery treatment.	RCTs = 5 N = 179	Population: patients with lateral epicondylitis Intervention: open, percutaneous or arthroscopic intervention Comparison: open, percutaneous or arthroscopic intervention Study type: RCTs	Quality assessment completed but criteria and explanation unclear.	Performance bias is present as the surgical interventions and postoperative protocols were not identical for all studies. There was a wide heterogeneity in surgical procedures and outcome measures used in the included studies.	There are no clinically significant differences between the 3 surgical techniques (open, arthroscopic, and percutaneous) in terms of functional outcome (DASH), pain intensity (VAS), and patient satisfaction at 1-year follow-up in subjects with LE.
**Lucado et al.** **(2018)** [42] **USA**	To determine whether joint mobilisations are effective in improving pain, grip strength, and disability in adults with LET.	CINAHL, PubMed, and PEDro. No starting search date. Last search date in June 2017. Search terms defined. No limitations described. Evidence of reference and hand searching. Eligibility criteria: RCTs, English, mobilisation or manipulation, and lateral elbow tendinopathy.	RCTs = 6 (3) N = 461 (205)	Population: patients with lateral epicondylitis Intervention: MWM, manipulation, mobilisation Comparison: US, corticosteroid, placebo injection, traditional treatment Study type: RCTs	Clear quality appraisal of the studies.	There were very few studies with similar research design, outcomes, or follow-up time periods.	There is compelling evidence that joint mobilisations have a positive effect on both pain and/or functional grip scores across all time frames compared to control groups in the management of LET.
**Lin et al.** **(2018)** [43] **Taiwan**	To explore the effectiveness of botulinum toxin compared with non-surgical treatments in patients with lateral epicondylitis.	PubMed, Scopus, Embase, and Airity Library. No starting search date. Last search date in February 2017. Search terms defined. No limitations described. Evidence of reference and hand searching. Eligibility criteria: RCTs, English and Chinese, lateral epicondylitis, and botulinum toxin.	RCTs = 6 N = 310	Population: patients with lateral epicondylitis Intervention: botulinum toxin injection Comparison: placebo injection Study type: RCTs	Clear quality appraisal of the studies.	Not all the RCTs documented other possible adverse events, including infection, tingling sensation, and tenderness related to injections.	When treating lateral epicondylitis, botulinum toxin was superior to placebo and could last for 16 weeks. Corticosteroid and botulinum toxin injections were largely equivalent, except the corticosteroid injections were better at pain relief in the early stages and were associated with less weakness in grip in the first 12 weeks.
**Navarro-Santana et al.** **(2020)** [44] **Spain**	To evaluate the effect of dry needling alone or combined with other treatment interventions on pain, related-disability, pressure pain sensitivity, and strength in people with lateral epicondylalgia of musculoskeletal origin.	MEDLINE, CINAHL, PubMed, PEDro, Cochrane Library, SCOPUS, and Web of Science databases from their inception to 5 April 2020.	RCTs = 7 N = 320	Population: patients with lateral epicondylitis Intervention: trigger-point dry needling Comparison: low-level laser, manipulation, ultrasound, ESWT Study type: RCTs	Clear quality appraisal of the studies.	The number of included trials was small (n = 7). Additionally, needling interventions were applied with different dosages. Another potential limitation is the heterogeneity and imprecision of the results of some of the trials.	The current meta-analysis found low evidence supporting the application of dry needling for the treatment of lateral epicondylalgia of musculoskeletal origin; however, some questions remain to be elucidated in future studies.
**Karanasios et al.** **(2021)** [45] **Greece**	To evaluate the effectiveness of exercise compared with other conservative interventions in the management of LET.	MEDLINE, PubMed, CINAHL, EMBASE, PEDro, ScienceDirect, Cochrane Library, and Grey literature databases were systematically searched from inception to November 2019.	RCTs = 30 N = 2123	Population: patients with lateral epicondylitis Intervention: exercise Comparison: exercise, manipulation, corticosteroid, wait-and-see Study type: RCTs	Clear quality appraisal of the studies.	Despite including 30 studies with over 2000 participants, there were no studies with a low risk of bias.	Low and very low certainty evidence suggests exercise is effective compared with passive interventions with or without invasive treatment in LET, but the effect is small.

**Table 2:** Treatments and terminology used to define LEP.

Review Year Country	TERMINOLOGY ADOPTED	TREATMENT
**van der Windt et al.** **(1999)** [21] **The Netherlands**	Tennis elbow, lateral epicondylitis, lateral epicondylalgia, epicondylalgia	Ultrasound (US)
**Struijs et al.** **(2002)** [22] **Australia**	Tennis elbow, lateral epicondylitis	Orthotic devices
**Green et al.** **(2002)** [23] **Australia**	Tennis elbow, epicondylalgia	Acupuncture
**Borkholder et al.** **(2004)** [24] **USA**	Tennis elbow, lateral epicondylitis	Splinting
**Trudel et al.** **(2004)** [25] **Canada**	Tennis elbow, epicondylalgia	US, acupuncture, rebox, wait-and-see, exercise, mobilisation, ionisation, laser, pulsed electromagnetic field
**Buchbinder et al.** **(2004)** [26] **Australia**	Tennis elbow	ESWT
**Bisset et al.** **(2005)** [27] **Australia**	Tennis elbow, lateral epicondylitis, extensor carpi radial tendinitis, epicondylalgia	Laser, ESWT, manipulation, mobilisation, exercise tape, orthotics, acupuncture, iontophoresis
**Herd** **(2008)** [28] **The Netherlands**	Tennis elbow, lateral epicondylitis, lateral epicondylalgia	Manipulative therapy, Cyriax, mobilisation with movement (MWM)
**Barr et al.** **(2009)** [29] **UK**	Lateral epicondylitis	Corticosteroid injection, corticosteroid injection with exercise, manipulation
**Coombes et al.** **(2010)** [30] **Australia**	Tennis elbow, lateral epicondylitis, lateral epicondylalgia, epicondylalgia, lateral elbow pain	Corticosteroid injection, corticosteroid injection with exercise
**Tumilty et al.** **(2010)** [31] **New Zealand**	Tennis elbow, lateral epicondylitis, extensor carpi radial tendinitis, epicondylalgia	LLLT
**Kalichman et al.** **(2011)** [32] **Israel**	Tennis elbow, lateral epicondylitis	Botulinum toxin A injection
**Buchbinderat al.** **(2011)** [33] **Australia**	Tennis elbow, lateral epicondylitis	“Open” surgery, percutaneous surgery
**Raman et al.** **(2012)** [34] **Canada**	Tennis elbow, lateral epicondylitis, lateral elbow tendinopathy, epicondylosis, lateral epicondylar tendinopathy	Resistance training (isometric, eccentric, concentric, isokinetic)
**Ahmad et al.** **(2013)** [35] **UK**	Lateral epicondylitis, lateral elbow tendinopathy, chronic elbow tendinosis	PRP
**Pattanittumet al.** **(2013)** [36] **Australia**	Tennis elbow, lateral epicondylitis	NSAIDs
**Cullinane et al.** **(2014)** [37] **New Zealand**	Tennis elbow, lateral epicondylalgia	Eccentric exercise, eccentric exercise with other therapies
**Tang et al.** **(2015)** [38] **China**	Tennis elbow, lateral epicondylitis	Acupuncture, electro-acupuncture
**Tsikopouloset al.** **(2016)** [39] **Greece**	Tennis elbow, lateral epicondylitis, lateral elbow tendinopathy	Autologous whole-blood intervention, corticosteroid injection
**Mattie et al.** **(2017)** [40] **USA**	Tennis elbow, lateral epicondylitis, common extensor tendinosis	Percutaneous tenotomy
**Burn et al.** **(2017)** [41] **USA**	Tennis elbow, lateral epicondylitis	Open, percutaneous, or arthroscopic intervention
**Lucado et al.** **(2018)** [42] **USA**	Lateral epicondylitis, lateral epicondylalgia	MWM, manipulation, mobilisation
**Lin et al.** **(2018)** [43] **Taiwan**	Tennis elbow, lateral epicondylitis	Botulinum toxin injection
**Navarro-Santana et al.** **(2020)** [44] **Spain**	Tennis elbow, lateral epicondylitis, lateral elbow tendinopathy	Trigger-point dry needling
**Karanasios et al.** **(2021)** [45] **Greece**	Tennis elbow, lateral epicondylitis, lateral elbow tendinopathy, epicondylalgia	Eccentric exercise, isometric exercise, corticosteroid, manipulation

**Table 3:** RoBis, risk of bias for systematic reviews [18].

**Review** **Year** **Country**	**PHASE 2**				**PHASE 3**
	1. Study Eligibility Criteria	2. Identification and Selection of Studies	3. Data Collection and Study Appraisal	4. Synthesis and Findings	Risk of Bias in the Review
**van der Windt et al.** **(1999)** [21] **The Netherlands**	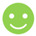	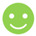	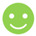	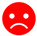	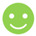
**Struijs et al.** **(2002)** [22] **Australia**	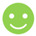	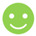	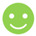	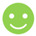	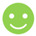
**Green et al.** **(2002)** [23] **Australia**	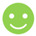	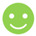	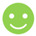	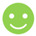	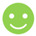
**Borkholder et al.** **(2004)** [24] **USA**	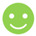	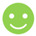	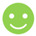	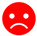	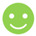
**Trudel et al.** **(2004)** [25] **Canada**	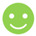	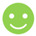	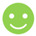	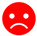	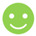
**Buchbinder et al.** **(2004)** [26] **Australia**	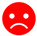	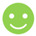	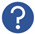	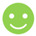	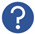
**Bisset et al.** **(2005)** [27] **Australia**	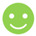	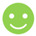	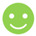	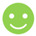	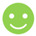
**Herd** **(2008)** [28] **The Netherlands**	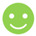	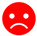	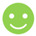	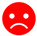	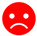
**Barr et al.** **(2009)** [29] **UK**	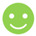	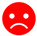	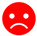	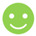	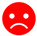
**Coombes et al.** **(2010)** [30] **Australia**	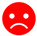	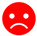	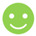	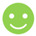	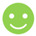
**Tumilty et al.** **(2010)** [31] **New Zealand**	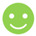	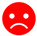	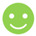	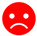	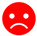
**Kalichman et al.** **(2011)** [32] **Israel**	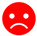	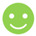	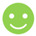	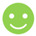	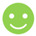
**Buchbinder at al.** **(2011)** [33] **Australia**	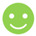	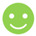	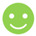	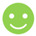	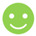
**Raman et al.** **(2012)** [34] **Canada**	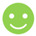	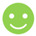	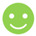	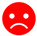	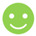
**Ahmad et al.** **(2013)** [35] **UK**	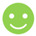	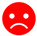	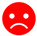	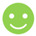	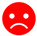
**Pattanittum et al.** **(2013)** [36] **Australia**	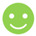	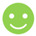	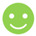	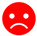	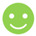
**Cullinane et al.** **(2014)** [37] **New Zealand**	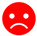	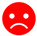	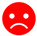	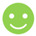	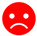
**Tang et al.** **(2015)** [38] **China**	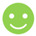	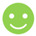	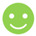	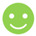	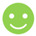
**Tsikopoulos et al.** **(2016)** [39] **Greece**	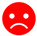	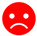	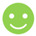	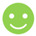	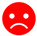
**Mattie et al.** **(2017)** [40] **USA**	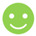	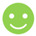	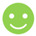	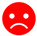	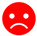
**Burn et al.** **(2017)** [41] **USA**	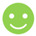	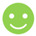	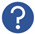	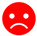	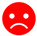
**Lucado et al.** **(2018)** [42] **USA**	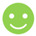	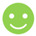	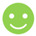	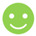	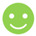
**Lin et al.** **(2018)** [43] **Taiwan**	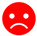	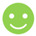	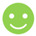	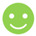	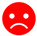
**Navarro-Santana et al.** **(2020)** [44] **Spain**	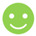	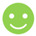	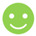	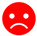	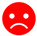
**Karanasios et al.** **(2021)** [45] **Greece**	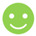	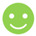	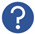	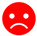	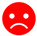

## Data Availability

Raw data are available upon request.

## Supplementary Materials

The following supporting information can be downloaded at: https://www.mdpi.com/article/10.3390/healthcare10061095/s1, Figure S1: LED-APP: Framework of Lateral Elbow Disorders Approach.

## Appendix A

**Table A1:** Literature search strategy.

The following databases were searched • Cochrane Library • Medline		
Search engines used: • PubMed Clinical Queries—Systematic Reviews • Cochrane Review • Epistemonikos		
Pubmed Clinical Queries Search Strategy Tennis elbow [MeSH Term] OR Tennis elbow OR Lateral epicondylitis OR Lateral epicondylalgia OR Lateral elbow tendinopathy	Cochrane Review Search Strategy Tennis elbow OR Lateral epicondylitis OR Lateral epicondylalgia OR Lateral elbow tendinopathy	Epistemonikos Search Strategy Tennis elbow OR Lateral epicondylitis OR Lateral epicondylalgia OR Lateral elbow tendinopathy
