# Supplementary figures and images for: Treatment, Diagnostic Criteria and Variability of Terminology for Lateral Elbow Pain: Findings from an Overview of Systematic Reviews
Source: Healthcare (Basel). 2022 Jun 14;10(6):1095. doi: 10.3390/healthcare10061095 (PMC9222841; doi:10.3390/healthcare10061095)

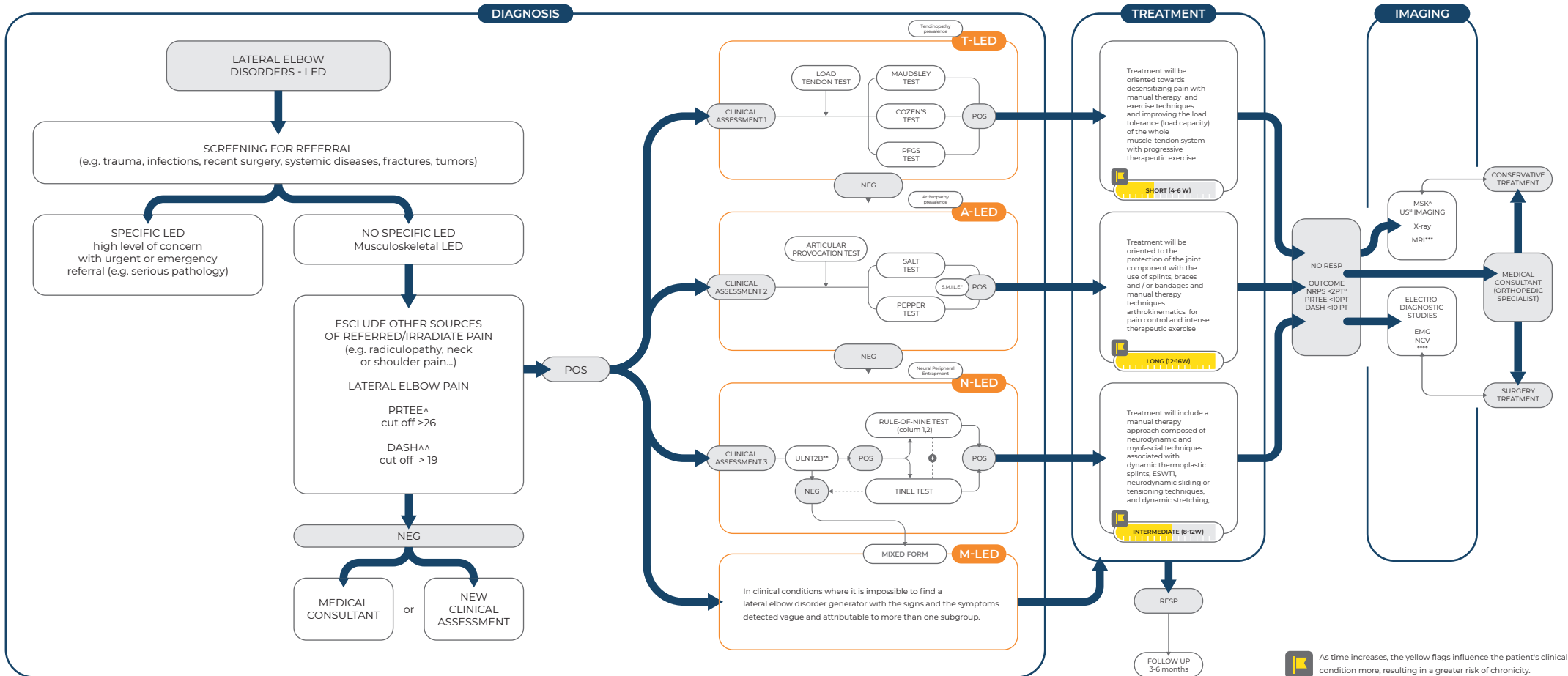

Supplement: Supplementary file 1 [file healthcare-10-01095-s001.zip › healthcare-1708159-supplementary.pdf]
